# Artificial Intelligence-Driven Approaches to Endoscopic Gastric Cancer Detection: Current Progress and Future Directions

**DOI:** 10.7759/cureus.81194

**Published:** 2025-03-25

**Authors:** Abdul Haseeb Hasan, Muhammad Ali Abid, Nida Waheed, Taimoor Wajid

**Affiliations:** 1 Medicine, Mayo Hospital, Lahore, PAK

**Keywords:** ai-assisted detection, artificial intelligence (ai), cancer, convolutional neural networks (cnns), deep learning, early gastric cancer (egc), endoscopic imaging, endoscopy, gastric cancer, machine learning (ml)

## Abstract

Gastric cancer (GC) is one of the most prevalent and lethal cancers globally, with early detection being crucial for improving patient survival rates. Endoscopy remains the primary diagnostic tool for gastric cancer, but its accuracy is highly dependent on the endoscopist's skill. Misdiagnosis rates, caused by factors such as incomplete lesion recognition and operator errors, remain a significant challenge. Artificial intelligence (AI), particularly deep learning models like convolutional neural networks (CNNs), has shown substantial potential in enhancing the detection of gastric cancer during endoscopic procedures. AI-driven systems have demonstrated the ability to improve diagnostic accuracy, reduce missed lesions, and standardize assessments, regardless of endoscopist experience. Studies have highlighted AI's effectiveness, with some models achieving diagnostic accuracy comparable to senior practitioners. However, challenges such as data quality, false positives/negatives, geographical biases, and regulatory barriers need to be addressed for broader clinical implementation. Despite these limitations, ongoing advancements in AI technology, coupled with multinational research and improved dataset diversity, hold promise for improving the early detection of gastric cancer, enhancing patient outcomes, and optimizing clinical workflows.

## Editorial

Introduction

Gastric cancer (GC) remains one of the most prevalent and lethal malignancies worldwide, ranking among the top five cancers in incidence and as the third leading cause of cancer-related mortality [1,2]. In 2020, gastric cancer was diagnosed in approximately 1.1 million people worldwide, with 720,000 cases in males and 370,000 in females [3]. The disease also led to 770,000 deaths that year [3]. If current incidence rates persist, the number of new cases is expected to rise by 62%, reaching 1.8 million by 2040, while mortality could increase to 1.3 million [3]. Its development is influenced by a combination of dietary, environmental, and genetic factors and is characterized by aggressive and heterogeneous biological behaviour [4]. The prognosis of GC is largely dependent on the stage at diagnosis, with patients in advanced stages facing a median survival of less than a year and a five-year survival rate below 20% [1,4]. In contrast, early gastric cancer (EGC), defined as tumor involvement confined to the mucosa and submucosa regardless of lymph node metastasis, has a significantly improved prognosis [4]. When detected and treated in its early stages, EGC has a five-year survival rate exceeding 90% [1,2,4]. Consequently, early diagnosis and timely intervention are crucial in reducing GC-related mortality [1,2,4].

Endoscopy remains the primary modality for diagnosing both GC and EGC, yet its diagnostic accuracy is highly dependent on the experience and proficiency of the endoscopist [1,4]. Studies have highlighted notable rates of misdiagnosis, with a meta-analysis including studies from 22 countries revealing that 622 out of 6,961 GC cases were underdiagnosed, reflecting an 8.94% misdiagnosis rate [4]. Similarly, another study reported that 7.2% of patients with GC had been misdiagnosed at an endoscopy performed within the previous year, with 73% of these cases attributed to endoscopist errors [2]. Contributing factors to missed diagnoses include inadequate lesion recognition, incomplete mapping of gastric anatomy, and biopsy sampling errors [4]. In response to these limitations, efforts have been made to improve diagnostic precision, including the adoption of advanced imaging techniques such as narrow band imaging (NBI), magnifying endoscopy (ME), blue laser imaging (BLI), and linked color imaging (LCI) [4]. While these modalities enhance lesion detection, their widespread implementation remains constrained by resource availability and the lengthy learning curve required for mastery [4]. This underscores the need for adjunctive tools that can assist endoscopists in achieving accurate and efficient diagnoses [4].

Artificial intelligence (AI) has emerged as a transformative technology in medical imaging, offering the potential to enhance diagnostic accuracy in various fields, including ophthalmology, dermatology, and oncology [4]. Within gastroenterology, AI applications have demonstrated success in detecting colorectal tumors, esophageal squamous cell carcinoma, and hepatic lesions [4]. Machine learning, a fundamental component of AI, allows for the iterative refinement of diagnostic models through data processing, while deep learning, particularly convolutional neural networks (CNNs), has proven highly effective in image analysis [1,2,4]. CNNs, designed to mimic neural processing in the human brain, are particularly well-suited for medical image recognition and have shown promise in improving diagnostic outcomes in endoscopic evaluations [4].

Since AI-based approaches for detecting gastrointestinal tumors are steadily evolving, this editorial aims to explore the application of artificial intelligence in the screening and diagnosis of gastric cancer through endoscopy. By examining AI-driven advancements, we highlight AI's potential to enhance detection accuracy and reduce diagnostic errors in endoscopic evaluations.

AI-enhanced endoscopy

AI has demonstrated substantial efficacy in enhancing the detection of GC through endoscopy, addressing key challenges associated with traditional diagnostic methods. White light endoscopy (WLE) remains the most widely used technique for GC screening, but its accuracy is highly dependent on the skill and experience of the endoscopist [1,2,4]. Studies have shown that AI-assisted systems can significantly improve diagnostic performance by reducing the rate of missed lesions and standardizing assessments across varying levels of expertise [4]. In China, a large-scale study analyzing 29,809 endoscopic images from 8,947 patients found that an AI-based system achieved an overall accuracy of 93.5% in diagnosing EGC, a result comparable to that of senior endoscopists [4]. Moreover, when endoscopists used AI assistance, their diagnostic accuracy increased to 94.9% for senior practitioners and 94.1% for junior practitioners, demonstrating AI's role as a valuable diagnostic aid [4].

Several deep convolutional neural network (DCNN)-based systems have been developed to enhance EGC detection [4]. In a study conducted in China, Wu et al. designed an AI model that outperformed endoscopists across all experience levels, achieving 91.0% specificity, 94.0% sensitivity, and 92.5% accuracy [2]. Additionally, the system incorporated an automated function for blind spot monitoring, ensuring a comprehensive examination of the gastric mucosa [2]. Similarly, in another study conducted in China, a DCNN model was trained using 8,600 benign images and 3,400 EGC images [4]. It demonstrated high accuracy (92.07%), specificity (92.05%), and sensitivity (92.08%) in external validation, surpassing the performance of less experienced endoscopists [4]. In Japan, a CNN-based diagnostic system was developed utilizing a dataset of 13,584 endoscopic images of gastric cancer [4]. When tested on an independent dataset of 2,296 stomach images from 69 patients with gastric cancer lesions, the model demonstrated high diagnostic performance, achieving an overall sensitivity of 92.2% [4]. In a study conducted in South Korea, a CNN model trained on 10,181 WLE images was implemented, which attained a 100% detection rate for EGC with an overall accuracy of 60.83%, highlighting the need for further refinement in classification capabilities [4].

Other AI-driven models have also yielded promising results in independent trials. In China, Zhang et al. developed a diagnostic system incorporating ResNet34 and DeepLabv3 architectures [5]. The trained CNN was tested on a dataset of 1,091 images, achieving a specificity of 91.2%, sensitivity of 36.8%, accuracy of 78.7%, positive predictive value of 55.4%, and negative predictive value of 82.9%. Notably, its specificity and positive predictive value (91.2% and 55.4%, respectively) were significantly higher than those of endoscopists (86.7% and 41.7%, respectively) [5]. Similarly, other studies from China, South Korea and Japan have validated AI systems with accuracy rates exceeding 90%, reinforcing AI’s ability to support clinical decision-making [4].

Challenges, limitations and future directions

Despite the remarkable advancements in AI for endoscopic gastric cancer detection, several challenges and limitations hinder its widespread clinical adoption. One major issue is the reliance on retrospective studies, which often exclude low-quality images containing common clinical scenarios such as bleeding, mucus, and halos [1,4]. As a result, AI models may perform better in research settings than in real-world applications [1,4]. To improve generalizability, prospective studies incorporating diverse and lower-quality images should be conducted to better simulate real clinical environments.

The performance of AI depends heavily on the quality and diversity of training datasets, emphasizing the need for standardized quality assurance guidelines in endoscopy [1]. Poorly performed examinations, operator-related issues, and suboptimal imaging conditions can lead to AI-related diagnostic errors [1]. Raising awareness and improving endoscopic technique standards are crucial for maximizing AI's effectiveness.

Another limitation is the persistent occurrence of false-positive and false-negative results, which may stem from inadequate differentiation between cancerous and non-cancerous lesion features [4]. This issue can be mitigated by expanding the training datasets with more annotated images and incorporating video-based learning to enhance AI's ability to recognize subtle gastric abnormalities. Additionally, most AI models are developed using endoscopic images from a single manufacturer, leading to difficulties in recognizing images captured by different endoscope brands [4]. Future studies should validate AI systems across multiple imaging platforms to ensure broader applicability.

Geographic and population-related biases also present challenges. Many studies on AI-assisted endoscopy have been conducted within specific countries, particularly China, making it difficult to generalize findings to diverse populations with different genetic, environmental, and lifestyle factors [2,4,5]. Collaborative multinational research is essential to establish AI’s global relevance.

AI adoption is further complicated by the difficulty in its regulation, as the safety and efficacy of AI tools must be thoroughly assessed before they can be integrated into clinical workflows [1]. Moreover, AI's impact is most effective when applied to standardized diagnostic tasks, such as assessing gastric cancer risk based on endoscopic findings like atrophic gastritis and Helicobacter pylori infection [1]. However, the widespread adoption of AI-assisted risk stratification remains limited.

Looking forward, improving AI transparency and interpretability will be crucial for gaining physician confidence in AI-aided diagnoses [1]. AI also holds the potential for improving endoscopy training by assisting young endoscopists in recognizing early gastric cancer lesions [1]. Future AI applications may integrate lesion characterization with detection, enhancing diagnostic precision and reducing unnecessary biopsies of non-cancerous lesions.

Conclusion

In conclusion, AI has shown significant promise in enhancing the detection of gastric cancer, particularly early gastric cancer, through endoscopy. By improving diagnostic accuracy and reducing missed lesions, AI supports endoscopists across all levels of expertise. However, challenges such as data quality, false positives, and regulatory hurdles remain. With ongoing advancements and collaborative research, AI has the potential to greatly improve early detection, patient outcomes, and clinical efficiency in gastric cancer diagnosis.
